# Impact of an Antimicrobial Stewardship Program on the Incidence of Carbapenem Resistant Gram-Negative Bacilli: An Interrupted Time-Series Analysis

**DOI:** 10.3390/antibiotics10050586

**Published:** 2021-05-16

**Authors:** Teresa López-Viñau, Germán Peñalva, Lucrecia García-Martínez, Juan José Castón, Montserrat Muñoz-Rosa, Ángela Cano, Manuel Recio, José Miguel Cisneros, Elena Pérez-Nadales, José Rumbao Aguirre, Elena García-Martínez, Inmaculada Salcedo, José Ramón del Prado, Carmen de la Fuente, Luis Martínez-Martínez, Irene Gracia-Ahufinger, Julián Torre-Cisneros

**Affiliations:** 1 Pharmacy Unit, Reina Sofia University Hospital, 14004 Cordoba, Spain; tessa220590@gmail.com (T.L.-V.); lucrecia.garcia.sspa@juntadeandalucia.es (L.G.-M.); joser.prado.sspa@juntadeandalucia.es (J.R.d.P.); 2 Infectious Diseases Unit, Reina Sofia University Hospital, Maimonides, Biomedical Research Institute of Cordoba (IMIBIC), University of Cordoba (UCO), 14004 Cordoba, Spain; juanj.caston.sspa@juntadeandalucia.es (J.J.C.); cayuam@hotmail.com (Á.C.); manuel.recio.sspa@juntadeandalucia.es (M.R.); elena.pereznadales@imibic.org (E.P.-N.); julian.torre.sspa@juntadeandalucia.es (J.T.-C.); 3 Department of Infectious Diseases, Microbiology and Preventive Medicine, Institute of Biomedicine of Seville, Virgen del Rocio University Hospital, 41013 Seville, Spain; german.penalva@gmail.com (G.P.); josem.cisneros.sspa@juntadeandalucia.es (J.M.C.); 4 Microbiology Unit, Reina Sofia University Hospital, IMIBIC, Department of Agricultural Chemistry, Edafology and Microbiology, University of Cordoba, 14004 Cordoba, Spain; montserrat.munoz.sspa@xn--juntadeandaluca-ipb.es (M.M.-R.); luis.martinez.martinez.sspa@juntadeandalucia.es (L.M.-M.); 5 Hospital Management, Reina Sofia University Hospital, 14004 Cordoba, Spain; josem.rumbao.sspa@juntadeandalucia.es (J.R.A.); elena.garcia.martinez.sspa@juntadeandalucia.es (E.G.-M.); 6 Preventive Medicine Unit, Reina Sofia University Hospital, 14004 Cordoba, Spain; minmaculada.salcedo.sspa@juntadeandalucia.es; 7 Intensive Care Unit, Reina Sofia University Hospital, 14004 Cordoba, Spain; carmen.fuente.sspa@juntadeandalucia.es

**Keywords:** antimicrobial stewardship program, carbapenems, antimicrobial resistance, carbapenem-resistant Enterobacteriaceae, carbapenemases

## Abstract

Carbapenem-resistant Gram-negative bacilli (CR-GNB) are a critical public health threat, and carbapenem use contributes to their spread. Antimicrobial stewardship programs (ASPs) have proven successful in reducing antimicrobial use. However, evidence on the impact of carbapenem resistance remains unclear. We evaluated the impact of a multifaceted ASP on carbapenem use and incidence of CR-GNB in a high-endemic hospital. An interrupted time-series analysis was conducted one year before and two years after starting the ASP to assess carbapenem consumption, CR-GNB incidence, death rates of sentinel events, and other variables potentially related to CR-GNB incidence. An intense reduction in carbapenem consumption occurred after starting the intervention and was sustained two years later (relative effect −83.51%; 95% CI −87.23 to −79.79). The incidence density of CR-GNB decreased by −0.915 cases per 1000 occupied bed days (95% CI −1.743 to −0.087). This effect was especially marked in CR-*Klebsiella pneumoniae* and CR-*Escherichia coli*, reversing the pre-intervention upward trend and leading to a relative reduction of −91.15% (95% CI −105.53 to −76.76) and −89.93% (95% CI −107.03 to −72.83), respectively, two years after starting the program. Death rates did not change. This ASP contributed to decreasing CR-GNB incidence through a sustained reduction in antibiotic use without increasing mortality rates.

## 1. Introduction

The increase in carbapenem-resistant Gram-negative bacilli (CR-GNB), in particular Enterobacteriaceae, is a growing public health problem worldwide and has led to the development of coordinated and comprehensive global action plans [1,2]. Infections caused by CR-GNB present few therapeutic options due to co-resistance to multiple antimicrobial groups, leading to a very high mortality rate that exceeds 50% in some studies [3]. In Spain, the incidence of carbapenem-resistant *Klebsiella pneumoniae* (CR-Kp) remains especially worrisome, showing a significant increasing trend from 2015 to 2019 [4]. Carbapenem use is among the most significant factors favoring the development of carbapenem-resistant enterobacteria, worsening hospital outbreaks, and/or endemics [5,6]. To tackle this problem, the implementation of antimicrobial stewardship programs (ASPs) has encouraged the promotion of the prudent use of antibiotics and minimize the emergence and spread of antibiotic resistance. Some educational and interventional measures have proven effective in reducing antimicrobial consumption and resistance [7,8,9,10,11,12]. However, the design quality of most studies evaluating ASPs is low, and only a small number of studies report microbiological data, with great heterogeneity in design and outcome endpoints. Consequently, it is not possible to reach a conclusion on changes in antibiotic resistance, thus making further studies necessary to analyze this association [13,14,15]. Since 2012, our hospital has an ongoing institutional ASP (PROA) aimed at optimizing antimicrobial use. In 2014, a Program for the Validation of Restricted-Use Antibiotics (PROVAUR) aimed at carbapenems was launched as a strategic action within the framework of PROA in response to a high rate of KPC-3-producing *K. pneumoniae* (KPC-Kp) suffered by our center [16,17]. In this work, we study the efficacy and safety of PROVAUR to optimize the use of carbapenems and the impact of these measures on carbapenem resistance in all adult inpatient areas of a tertiary-care hospital with a high incidence of these microorganisms. We hypothesized that this program would lead to a reduction in carbapenem consumption and would reduce the incidence of CR-GNB, particularly the endemic CR-Kp in our hospital, without increasing mortality rates.

## 2. Results

During the study period, 125,847 adult patients were admitted to the hospital, 48.2% of whom were males (Table S1). Since the start of the PROVAUR, a total number of 1359 face-to-face educational interviews were conducted between the prescribers and the specialists from the antimicrobial stewardship team (see“Intervention” in the Materials and Methods section).

### 2.1. Antimicrobial Consumption

**Figure 1:**
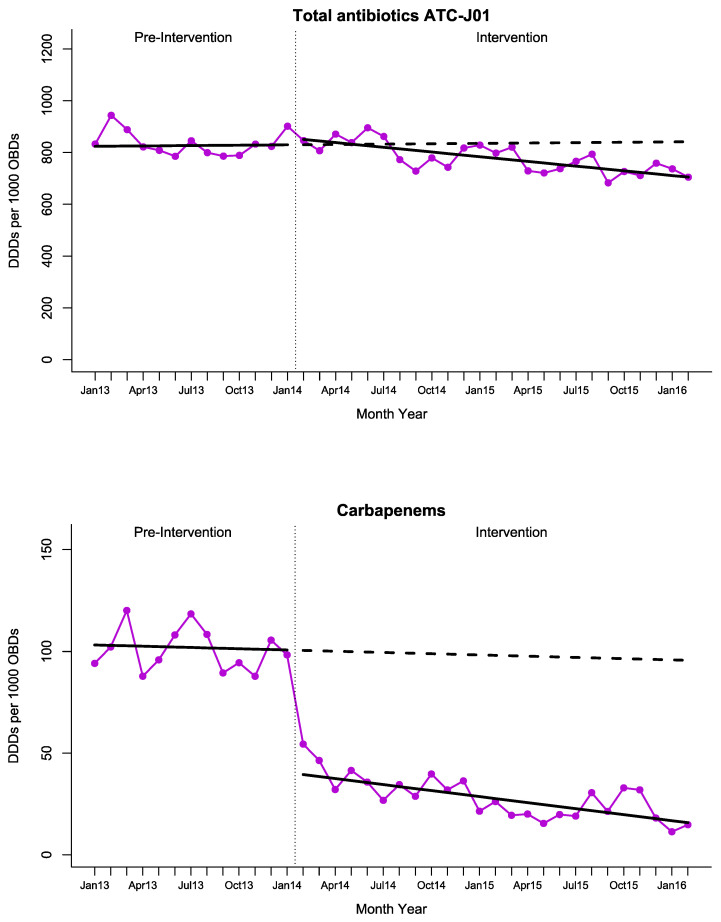
Interrupted time series analysis of the trends in ATC group J01 (antibacterials for systemic use) and carbapenem (meropenem/imipenem) consumption observed before and after the implementation of the antimicrobial stewardship program. Solid purple line: antibiotic consumption time series. Solid black lines: observed trend during the pre-intervention and intervention periods. Dashed black line: counterfactual (expected) trend after the intervention according to the pre-intervention values. DDDs: defined daily doses; OBDs: occupied bed days.

The implementation of PROVAUR was associated with a long-term reduction in carbapenem consumption and the ATC-J01 group (antibacterials for systemic use), with significant absolute and relative differences from the pre-intervention trend (Table 1, Figure 1). This reduction was especially marked in carbapenems, which showed a rapid absolute decrease of −60.32 defined daily doses (DDD) per 1000 occupied bed days (OBD) (95% CI −68.83 to −51.80; *p* < 0.0001) one month after the beginning of the intervention, whose effect was maintained over time until reaching a relative reduction of −83.51% (95% CI −87.23 to −79.79) at the end of the two-year intervention period. The ATC-J01 group also showed a significant change in trend during the intervention, with a relative decrease in consumption at two years of −16.22% (CI 95% −25.64 to −6.81). The joinpoint regression analysis is shown in Table S2 and Figure S1. The use of β-lactams/β-lactamase inhibitors changed from a downward pre-intervention trend to a sustained increase during the intervention period, while the consumption of third- and fourth-generation cephalosporins showed a stable trend during the intervention period after an upward pre-intervention trend. No differences between periods were observed in the consumption of quinolones and aminoglycosides at the end of the study (Table 1, Figure S2).

### 2.2. Carbapenem Resistant

**Figure 2:**
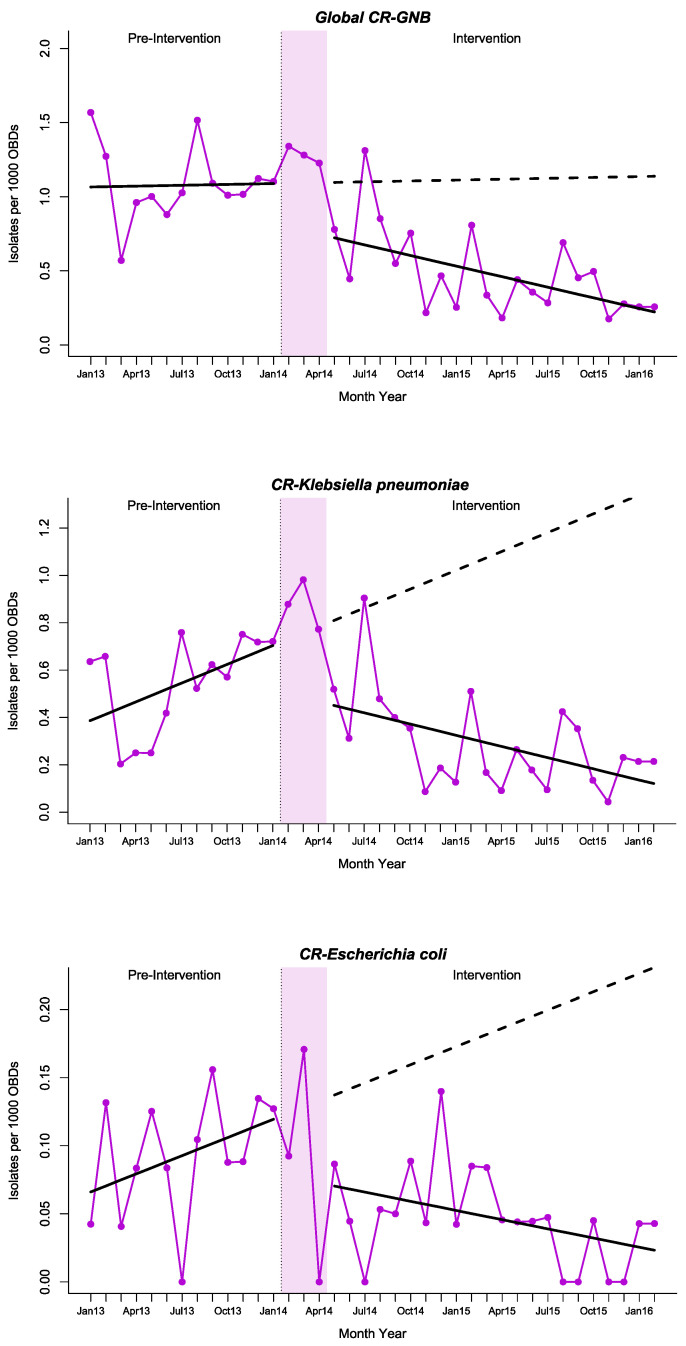
Interrupted time series analysis of changes in trends of incidence density of global carbapenem-resistant Gram-negative bacilli (CR-GNB), CR-*K. pneumoniae* and CR-*E. coli* observed before and after the intervention. Solid purple line: incidence density of CR-GNB time series. Solid black lines: observed trend during the pre-intervention and intervention periods. Dashed black line: counterfactual (expected) trend after the intervention according to the pre-intervention values. OBDs: occupied bed days.

The incidence density of CR-GNB decreased significantly, with a relative reduction of 80.46% (95% CI; −90.49 to −70.43) two years after starting the program, compared with the expected values in the absence of intervention. This effect was especially marked in *K. pneumoniae* and *Escherichia coli*, where the increasing trend in the pre-intervention period reverted towards an intense decreasing trend during the intervention (Table 2, Figure 2). Thus, CR-Kp showed a relative reduction of −91.15% (95% CI; −105.53 to −76.76) two years after starting the program, accounting for an absolute reduction of −1.244 cases per 1000 OBDs (−2.060 to −0.427). In CR-*E. coli*, a relative reduction of −89.93% (95% CI; −107.03 to −72.83) was observed, achieving an absolute decrease of −0.207 cases per 1000 OBDs (−0.376 to −0.039) at the end of the study period. The Joinpoint regression analysis is described in Table S3 and Figure S3. The proportion of CR-*Kp* and *CR-E. coli* per year is shown in Table 3 and Table S4, respectively. The incidence density of CR-*Pseudomonas aeruginosa* and CR-*Acinetobacter baumannii* showed a downward trend beginning in the pre-intervention period, presenting no changes associated with the intervention, while the incidence of the other Enterobacteriaceae remained steady during the study period (Table 2, Figure S4).

### 2.3. Impact on Mortality Rates for Sentinel Events

Regarding the program’s safety, the 14-day crude death rate remained stable throughout the study period, showing no significant changes related to the intervention (Table 4, Figure S5).

### 2.4. Changes in Healthcare during the Study Period

Infection control indicator trends remained steady. Thus, the proportion of hand-hygiene compliance did not present any change in trend during the study period (Table S5, Figure S6). Moreover, correct contact isolation was observed in all of indicated cases, and all hospital complexity indicators showed a stable trend, except for the number of solid-organ transplantations, which increased progressively throughout the study period (Figure S7).

### 2.5. Acceptance of the PROVAUR Program

Three hundred questionnaires were distributed to the physicians, of which 282 (94%) were completed. Of these, 278 (98.6%) were familiar with the program, 275 (97.5%) stated that it was useful, and 276 (97.9%) were satisfied with its implementation.

## 3. Discussion

Our results show the positive impact of a bundle of educational and restrictive measures to optimize carbapenem use and reduce the incidence of CR-GNB in a hospital with a high endemic of CR-Kp. Moreover, these measures had no effect on the mortality rate for sentinel events and were met with a high degree of acceptance by the prescribers. Carbapenem consumption reduced markedly after the intervention, and in parallel, a significant reduction in the rate of CR-GNB was observed, especially in CR-*K. pneumoniae* and CR-*E. coli*, thus supporting the initial hypothesis that a reduction in carbapenem pressure can contribute to reducing bacterial resistance to these antibiotics. As previously described [5,6], the overuse of carbapenems is one of the most significant risk factors favoring the spread of CR-Enterobacteriaceae. It is known that carbapenemase genes are transported by a small number of plasmids and transposons, which allows them to acquire successful multi-resistant platforms of rapid global expansion. When a resistance plasmid has been transferred and replicated in a new bacterial host, the existence of antimicrobials will produce an artificial selective pressure that fosters the onset of bacterial populations containing such resistance plasmids, thus providing high-risk clones the opportunity to become successful intestinal colonizers and cause infection. However, in the absence of antimicrobials, the resistance plasmid may be retained only temporarily by the bacterium if it is unstable within the host [18]. In our hospital, the successful ST512 *K. pneumoniae* clone caused an important outbreak from an imported case in 2012 and was the first outbreak to be reported in Spain caused by the KPC-3-producing ST512 *K. pneumoniae* clone, leading to a mortality of 30% [19]. Although several steps were taken, the outbreak evolved into an endemic situation. Considering the above, carbapenem overuse could facilitate the selection of CR-GNB in patients with frank colonization that is detectable in colonization studies or in selected minority populations with these agents. Based on the favorable outcomes of PROVAUR resulting from a previous study [9], in 2014, we decided to adapt the program to optimize carbapenem use, achieving a more than 80% reduction in consumption within two years of its implementation. Although there is sufficient evidence about the positive effect of ASPs on improving antimicrobial use [6,7,8,9,10,11,12,13], recent systematic reviews have found no strong evidence of the efficacy of ASPs in reducing antibiotic resistance in hospitals due to the small number of studies evaluating this relationship, as well as a large heterogeneity in study designs and outcome endpoints [13,14,15,20]. Indeed, many of these studies were conducted in a single hospitalization unit, and the correlations between consumption and resistance were sometimes contradictory. Giacobbe et al. [7] implemented an educational and semi-restrictive ASP (excluding emergency and ICU units), during which they achieved a significant reduction in both carbapenem consumption and the incidence of CR-Kp infections. In contrast, Faraone et al. [6] implemented an educational and restrictive carbapenem ASP in an internal medicine unit and found that carbapenem consumption was significantly reduced but not the incidence of CR-Kp infections, which remained unchanged. Our ASP was conducted in all adult hospitalization units, including the ICU, where patients’ stay has been considered a risk factor for the development of CR-GNB [5]. The most relevant finding of our study was the marked ecological impact observed in CR-Kp and CR *E. coli*. Regarding CR-*P. aeruginosa* and CR-*A. baumannii*, the time-series showed a previous decreasing trend not related to our intervention, possibly caused by the ecological change that the arrival of KPC brought about in the epidemiology of our hospital. However, it must be considered that the prevalence of antimicrobial resistance is a result of multiple other factors, including infection control practices, exposure to other antimicrobials, and patient comorbidities [5]. Because the possibility of ecological bias is inherent to our study design, we analyzed infection control measures and complexity indicators of hospital activity as potential confounding factors, which remained unchanged except for the number of solid-organ transplantations, which increased throughout the study period. Furthermore, we evaluated the consumption of other antibiotics whose use has been shown to influence the growth of CR-GNB [5]. No significant changes were observed except for the consumption of β-lactams/β-lactamase inhibitors, which increased during the intervention, probably because they were prescribed as an appropriate alternative to carbapenem use. To assess the impact on the overall antimicrobial use, we evaluated the consumption of the ATC-J01 group, which significantly decreased during the intervention, likely due to the intense reduction in carbapenem use, which counteracted and surpassed the compensatory increase in the use of β-lactams/β-lactamase inhibitors. These findings suggest that our bundle of interventions was successful in improving overall antimicrobial use in the hospital without triggering the ‘squeezing balloon’ phenomenon described in other ASPs due to the overuse of other antimicrobials [12,21]. The core activity of PROVAUR combines a restrictive measure with an educational one. The latest Cochrane systematic review on ASPs concluded that implementing both educational and restrictive interventions increases appropriate antibiotic use without increasing the mortality risk. However, some studies have reported that restrictive interventions could be unsafe due to a delay in the initiation of empirical antibiotic therapy and may also lead to negative effects on professional culture through deterioration in confidence and communication with clinicians [13]. Our program allows carbapenems to be dispensed until the expert of the antimicrobial team reviews the case during the day or by the next working day if it is a weekend, thus avoiding delays in antibiotic dose, which in many cases is paramount. In addition, due to the educational approach of our program, it has shown a high degree of acceptance by prescribers, with 97.9% of them being satisfied with its implementation. Besides the core activity aimed especially at carbapenems use and coinciding with the launching of the Institutional Program for the Prevention and Control of Healthcare-associated Infections and Antimicrobial Stewardship in Andalusia (PIRASOA) [22], our program also implemented other measures during the intervention period to optimize antimicrobial use (see “Intervention” in the Materials and Methods section). Specifically, antibiotic consumption indicators were implemented in each department, guidelines for empirical treatment and surgical prophylaxis were updated, training sessions were conducted with the antimicrobial team, and a program was carried out to validate antibiotic treatment duration. Our results suggest that the sustainability of these measures, alongside the main ongoing activity of targeting carbapenem use, has enabled us to maintain the decreasing consumption levels throughout the intervention period. 

This study had some limitations. First, this is a single-center study, which implies the need to confirm the reproducibility of our findings in other healthcare centers and in the pediatric population, which was not analyzed. Secondly, it is a retrospective, quasi-experimental study. Most of these studies use an uncontrolled before-after design, which is much more susceptible to bias and random error. Therefore, we used the interrupted time series (ITS) analysis which, after clinical trials, is considered the most robust design to study the impact of health interventions [23,24]. Our study could not include a feasible control group as the intervention was set in the entire hospital. We performed a joinpoint regression analysis to enhance the strength of the inference and to reinforce our outcomes [25]. Our results detected significant inflection points consistent with the results of the ITS analyses (Tables S2 and S3 and Figures S1, S3, S5 and S6). Finally, a cost-effectiveness analysis was not performed. Some strengths of our study are a large number of variables analyzed, and the reporting of clinical and microbiological outcomes since the review of the current literature has shown that very few ASP studies include these results [8,9,10,12]. Regarding mortality outcomes, reports about the safety of ASP programs targeting carbapenems are scarce. Recent studies have reported that these carbapenem-targeted programs do not increase mortality rates [12,26]. However, it should be noted that these studies were based on educational strategies only (audit and feedback), unlike our program, which incorporates a restrictive measure. Furthermore, these studies were conducted in settings without endemicity of carbapenemase-producing Enterobacteriaceae; thus, the impact in highly endemic areas is uncertain. In our study, we evaluated the program’s safety by analyzing the 14-day crude death rate of several events considered sentinel, as they are related to significant mortality rates and for which carbapenems are considered a therapeutic option. Our results showed that no changes occurred during the study period, so we consider the program a safe strategy. Given the substantial reduction in CR-GNB isolates reported in our study, it would be interesting in the future to assess whether this reduction is indeed associated with a reduction in mortality rates of infections due to these multidrug-resistant microorganisms, as would be expected. 

## 4. Materials and Methods

### 4.1. Study Design and Period

We performed a quasi-experimental, before-after study of ITS based on an ecological time-trend analysis, following the ORION statement. The study period included one year before (January 2013–January 2014) and two years after (February 2014–February 2016) the start of the intervention.

### 4.2. Study Setting and Population

The study was conducted at the Reina Sofia University Hospital (Cordoba, Spain), a 1000 bed tertiary-care teaching hospital with 32 intensive-care unit (ICU) beds and active solid-organ and hematopoietic stem-cell transplantation programs. No data from pediatric patients were recorded. Since June 2012, a high rate of KPC-Kp was detected after an infected patient was transferred from an Italian hospital. The index case and multiple other isolates representative of the initial outbreak and subsequent endemic period were characterized at the molecular level. The organisms corresponded to an ST512 clone and produced KPC-3, in addition to SHV-11 and TEM-1 [19]. The involved strain showed resistance to all tested β-lactams and carbapenems, fluoroquinolones, amikacin, tobramycin, and cotrimoxazole and presented variable susceptibility to fosfomycin, tigecycline, gentamicin, and colistin. The organism was susceptible to ceftazidime-avibactam, although a few isolates resistant to this combination have already been identified [27]. No other hospital outbreaks occurred during the study period. 

### 4.3. Intervention

PROVAUR was headed by a multidisciplinary team of 15 Infectious Diseases experts from different clinical units (validators), as well as a hospital pharmacist, who identified daily carbapenem prescriptions through the pharmacy’s computerized system, providing the antimicrobial stewardship team with a list of patients (name, clinical history number, bed, dose, and day of prescription). PROVAUR’s main activity has been described elsewhere [9] and was both restrictive (prescription form, Table 5) and educational, through a face-to-face interview between the patient’s attending physician and the specialist from the antimicrobial stewardship team, who jointly reviewed the case and discussed the appropriateness of the prescription and therapeutic alternatives. In addition to the core activity targeting carbapenems, this program also implemented additional measures to optimize antimicrobial use. Table 6 summarizes the activities carried out before the intervention and the bundle of interventional measures. PROVAUR was disseminated to health professionals in all clinical units through the hospital’s website, courses, and face-to-face sessions. To assess their level of acceptance, the professionals were invited to complete a voluntary and anonymous survey [9]. The program received institutional support, and its objectives were included in the annual agreement between each clinical unit with the hospital manager and the Andalusian Health Service. No other interventions were performed in the hospital during the study period. 

### 4.4. Variables and Definitions

The primary outcome variable was the incidence density of nosocomial infections or colonizations caused by CR-GNB per 1000 OBDs: *K. pneumoniae* and other Enterobacteriaceae, *A. baumannii*, and *P. aeruginosa*. The main explanatory variable was the consumption of the ATC group J01, carbapenems (meropenem/imipenem), β-lactams/β-lactamase inhibitors, third- and fourth-generation cephalosporins, quinolones, and aminoglycosides, which were recorded as DDD per 1000 OBDs and calculated according to the WHO’s Anatomical Therapeutic Chemical Classification System. Other variables potentially related to the incidence density of CR-GNB were also analyzed. Data on the average of the proportion of correct hand-hygiene compliance in each unit were collected, and handwashing was evaluated according to WHO recommendations [28]. The percentage of correct contact isolation in patients colonized/infected with extended-spectrum β-lactamase (ESBL) and CR-GNB was collected following the Centers for Disease Control and Prevention criteria [29], and indicators related to the complexity of the hospital’s activity were recorded as the “All-Patient Refined Diagnosis-Related Groups” (APR-DRGs) index [30], number of ICU OBDs, number of major surgical procedures, and number of transplants (solid-organ and hematopoietic stem-cell). The safety of the intervention was assessed by analyzing the all-cause crude death rate (deaths per 1000 OBDs) on day 14 after the diagnosis of sentinel events: bacteremia and nosocomial pneumonia (including ventilator-associated pneumonia) caused by ESBL-producing Enterobacteriaceae and *P. aeruginosa*. All the variables were recorded monthly.

### 4.5. Microbiological Definitions

A GNB was considered hospital-acquired when it was isolated from a sample obtained ≥ 48 h after a patient’s admission or in those cases when, even occurring in the first 48 h, the patient was hospitalized or had contact with the healthcare system during the previous 4 weeks. Recurrent isolation of the same microorganism was considered to represent a unique episode unless the culture was obtained 4 weeks after the last positive sample. Only isolates obtained from clinical samples were used to estimate infection/colonization incidence density. Isolates from rectal colonization studies were not considered, as these were only of epidemiological significance. During our study period, these studies were not protocolized hospital-wide and were only carried out in selected units during a specific period of time; therefore, they were not considered suitable for the objectives of this study. Identification and susceptibility testing were performed by commercial microdilution panels (WalkAway system, Dade MicroScan, Sacramento, CA, USA). The clinical category was classified according to the EUCAST breakpoints [31]. ESBL production was confirmed by disk-diffusion using discs (Bio-Rad Laboratories, Inc., Marnes-la-Coquette, France) of cefotaxime, ceftazidime, and cefepime with and without clavulanic-acid in all cases. Carbapenemase production was confirmed by either a commercial PCR assay (Xpert^®^ Carba-R, Cepheid Inc., Sunnyvale, CA, USA) or an immunochromatography assay (KPC K-SeT, Coris BioConcept, Belgium and NG-Test CARBA 5, NG-Biotech, Guipry, France).

### 4.6. Statistical Analysis

To assess the effect of the intervention, we used an ITS analysis. We performed a longitudinal segmented regression with a generalized least squares approach to evaluate changes in level and/or trend after the intervention, adjusting for seasonality and detecting possible outliers. Autocorrelation was considered using autoregressive moving-average models. To select the most parsimonious models, we applied the Akaike Information Criterion. The normality of residuals was verified, and the autocorrelation patterns were validated through likelihood-ratio tests to determine the final model for each variable. To assess whether the changes in results were attributable to the intervention, we calculated absolute and relative differences between observed changes and estimated values that would have been expected with no intervention for a time point equivalent to 2 years after the ASP. To take into account the delay between the effect of antimicrobial use and resistance, we considered a 3-month phase-in period [32] in the ITS analysis of the incidence density of CR-GNB. Statistical analyses were performed using R software, version 3.6.1. Furthermore, we performed a joinpoint regression analysis [25] (Joinpoint Regression Program, version 4.7.0) to test the robustness of the ITS analysis, as well as to detect significant trend changes associated with the intervention on indicators of hospital complexity. A *p*-value < 0.05 (two-tailed) was considered significant.

## 5. Conclusions

In conclusion, our ASP, based on a bundle of educational and restrictive measures, has proven effective and safe in reducing antibiotic use and has contributed to a decrease in CR-GNB, especially CR-Kp, in the context of a hospital with a high incidence of these resistant microorganisms. Our study could be useful in hospitals where carbapenems need to be targeted, and an infectious disease expert is available for intervention.

## Figures and Tables

**Table 1:** Interrupted time-series analysis of changes in trends of antimicrobial consumption.

Outcomes	Regression Intercept	Pre-Intervention Trend	Change in Level ^a^	Change in Trend ^b^	Absolute Effect ^c^	Relative Effect (%) ^c^
**Total antibiotics** **(ATC-J01)**	823.7	0.472 (−2.999, 3.943)	26.72 (3.210, 50.22)	−6.531 (−9.696, −3.365)	−136.5 (−237.2, −35.94)	−16.22 (−25.64, −6.809)
**Carbapenems**	103.4	−0.203 (−1.221, 0.815)	−60.32 (−68.83,−51.80)	−0.783 (−1.749, 0.182)	−79.90 (−110.6, −49.13)	−83.51 (−87.23, −79.79)
**Third- and fourth-generation cephalosporins**	67.94	2.168 (0.690, 3.645)	5.205 (−8.088, 18.50)	−1.896 (−3.473, −0.318)	−42.19 (−88.98, 4.585)	−28.07 (−49.03, −7.106)
**β-lactams and β-lactamase inhibitors**	237.2	−4.049 (−4.981, −3.118)	49.06 (42.46, 55.65)	3.623 (2.777, 4.469)	139.6 (112.5, 166.8)	167.6 (63.81, 271.4)
**Quinolones**	162.1	−0.391 (−1.233, 0.451)	7.290 (−4.924, 19.50)	−1.217 (−2.134, −0.299)	−23.13 (−53.21, 6.953)	−15.71 (−32.83, 1.411)
**Aminoglycosides**	20.336	−0.304 (−0.862, 0.255)	4.617 (−0.302, 9.536)	0.044 (−0.567, 0.654)	5.711 (−12.08, 23.50)	64.94 (−292.1, 422.0)

Data are presented as monthly defined daily doses (DDDs) per 1000 occupied bed days (OBDs) with a 95% confidence interval unless otherwise specified. ^a^ Increase or decrease in the first month after the start of the intervention period with respect to the expected value. ^b^ Change in slope for the intervention period. ^c^ Absolute or percentage difference between the expected value according to the pre-intervention trend of antibiotic prescription and the trend two years after the start of the intervention.

**Table 2:** Interrupted time-series analysis of changes in trends of incidence density of carbapenem-resistant Gram-negative bacilli.

Outcomes	Regression Intercept	Pre-Intervention Trend	Change in Level ^a^	Change in Trend ^b^	Absolute Effect ^c^	Relative Effect (%) ^c^
**Global CR-GNB**	1.063	0.002 (−0.025, 0.028)	−0.349 (−0.629, −0.068)	−0.026 (−0.053, 0.002)	−0.915 (−1.743, −0.087)	−80.46 (−90.49, −70.43)
***Klebsiella*** ***pneumoniae***	0.359	0.026 (0.0007, 0.052)	−0.316 (−0.609, −0.022)	−0.042 (−0.070, −0.014)	−1.244 (−2.060, −0.427)	−91.15 (−105.5, −76.76)
**Other Enterobacteriaceae**	0.137	0.001 (−0.003, 0.005)	−0.053 (−0.094, −0.013)	−0.004 (−0.008, 0.0005)	−0.133 (−0.257, −0.008)	−75.11 (−85.64, −64.58)
***Escherichia coli***	0.061	0.004 (−0.0009, 0.009)	−0.060 (−0.121, 0.0006)	−0.007 (−0.012, −0.0008)	−0.207 (−0.376, −0.039)	−89.93 (−107.0, −72.83)
***Proteus*** ***mirabilis***	0.023	−0.0001 (−0.003, 0.003)	−0.006 (−0.044, 0.031)	−0.0002 (−0.003, 0.003)	−0.011 (−0.109, 0.088)	−60.07 (−279.2, 159.1)
***Klebsiella*** ***aerogenes/*** ***Enterobacter cloacae***	0.015	0.0004 (−0.002, 0.003)	−0.006 (−0.029, 0.017)	−0.0007 (−0.003, 0.002)	−0.022 (−0.096, 0.051)	−68.72 (−113.52, −23.92)
***Pseudomonas*** ***aeruginosa***	0.258	−0.011 (−0.019, −0.003)	0.079 (−0.002, 0.161)	0.008 (−0.0004, 0.017)	0.260 (0.0009, 0.520)	−146.5 (−263.2, −29.84)
***Acinetobacter*** ***baumannii***	0.333	−0.016 (−0.023, −0.010)	−0.025 (−0.088, 0.037)	0.014 (0.007, 0.020)	0.283 (0.089, 0.477)	−96.25 (−110.9, −81.64)

Data are presented as the monthly incidence of healthcare-associated carbapenem-resistant Gram-negative bacilli (CR-GNB) with a 95% confidence interval unless otherwise specified. ^a^ Increase or decrease in the first month after the start of the intervention period with respect to the expected value. ^b^ Change in slope for the intervention period. ^c^ Absolute or percentage difference between the expected value according to the pre-intervention trend of incidence density and the trend two years after the start of the intervention.

**Table 3:** Proportion of carbapenem-resistant *Klebsiella pneumoniae* per year.

Year	Number of *K. pneumoniae* Isolates	Number of CR-Producing *K. pneumoniae*	Resistance Proportion (%)
**2013**	222	142	64%
**2014**	249	146	58.6%
**2015**	160	57	35.6%
2016 *	27	10	37%

* Data for 2016 correspond only to the months included in the study period: January and February.

**Table 4:** Interrupted time-series analysis of changes in sentinel event survival rate.

Regression Intercept	Pre-Intervention Trend	Change in Level ^a^	Change in Trend ^b^	Absolute Effect ^c^	Relative Effect (%) ^c^
0.103	−0.004 (−0.009, 0.001)	−0.010 (−0.057, 0.036)	0.004 (−0.0006, 0.009)	0.105 (−0.061, 0.272)	−190.9 (−612.4, 230.6)

Data are presented as monthly crude death rate with a 95% confidence interval unless otherwise specified. ^a^ Increase or decrease in the first month after the start of the intervention period with respect to the expected value. ^b^ Change in slope for the intervention period. ^c^ Absolute or percentage difference between the expected value according to the pre-intervention trend of crude death rate and the trend two years after the start of the intervention.

**Table 5:** Institutionally approved guidelines for carbapenem use.

Empirical treatment of nosocomial infections in areas with an endemic or epidemic outbreak of infections due to Gram-negative bacilli sensitive to carbapenems and resistant to other therapeutic options; Empirical treatment of severe nosocomial infections that need to cover *Pseudomonas aeruginosa* or ESBL-producing Enterobacteriaceae after the failure of other valid therapeutic options; Targeted treatment of severe infections due to: (i) sensitive Gram-negative bacilli when there are no other therapeutic alternatives or they have failed; (ii) multi-resistant Gram-negative bacilli in which carbapenems are the most reasonable option; Neutropenic patients with severe nosocomial infection presenting a possible abdominal (including severe mucositis) or gynecological focus after the failure of other valid therapeutic options; Empirical treatment of severe community infections in patients with risk factors for ESBL; Targeted treatment of infections caused by ESBL-producing microorganisms without other reasonable options.

ESBL: extended-spectrum β-lactamase.

**Table 6:** Measures carried out before and during the program for the Validation of Guidelines for Restricted-Use Antibiotics (PROVAUR program).

Periods	Activity
Pre-intervention	Carbapenems were dispensed without standardized written justification of the indication; The pharmacy unit issued quarterly information concerning the profile of antibiotic use in each service, with an analysis of the results obtained for feedback and comparative evaluation; A report on antimicrobial and overall spending was presented twice a year at a meeting attended by the hospital medical director, clinical unit directors, and the antimicrobial team; Active “Bacteremia program” conducted by an infectious disease specialist in all cases of bacteremia, with recommendations for patient management and targeted treatment; A consultancy program with infectious disease specialists was available; Colonization work-up: Rectal swab culture at admission and weekly in high-risk wards (ICU and hematology); Contact isolation of patients infected/colonized with CR-GNB and ESBL-producing Enterobacteriaceae.
Intervention	PROVAUR was authorized by the institution, and all services were informed about the program, which accepted the intervention and designated a departmental-level program manager; PROVAUR was described to each clinical unit in educational sessions; The Pharmacy and Therapeutics Committee updated guidelines for carbapenem use based on the local epidemiology and review of best available evidence by infectious disease specialists and informed all prescribers; A carbapenem prescription form for approved indications and personal prescription validation interview were developed; Each carbapenem prescription was validated on the next working day, and a personal prescription validation interview was conducted at the bedside by a member of the PROVAUR team; The pharmacy unit was contacted by phone or e-mail of the validation to continue dispensing carbapenems. Non-validated antimicrobials were replaced by alternative treatments following the recommendation by specialists; Antibiotic consumption indicators were implemented in each clinical unit; Quarterly and bi-annual information on overall consumption and profile of antibiotic use was maintained; Guidelines for empirical treatment and surgical prophylaxis were updated; An intervention program for the duration of antimicrobial treatment (PROVATEM) in all hospitalization units was implemented by the PROVAUR team; Training sessions were held monthly with the PROVAUR team for healthcare staff to review the most prevalent infectious syndromes in the hospital setting and the main aspects of antibiotic use; The bacteremia program and consultancy with infectious disease specialists were maintained; Colonization work-up in high-risk wards and contact isolation of patients with CR-GNB and ESBL-producing Enterobacteriaceae were maintained.

ICU: Intensive Care Unit; ESBL: extended-spectrum β-lactamase; CR-GNB: carbapenem-resistant Gram-negative bacilli.

## Data Availability

The data presented in this study are available on request from the corresponding author.

## Supplementary Materials

The following are available online at https://www.mdpi.com/2079-6382/10/5/586/s1, Table S1: Breakdown of monthly admitted patients. Table S2: Joinpoint regression analysis of antibiotic consumption. Table S3: Joinpoint regression analysis of the incidence density of carbapenem-resistant Gram-negative bacilli. Table S4: Proportion of carbapenem-resistant Escherichia coli per year. Table S5: Interrupted time-series analysis of changes in hand hygiene compliance. Figure S1: Joinpoint regression analysis of carbapenem/ATC-J01 consumption. Figure S2: Interrupted time-series analysis of changes in consumption of other antibiotics. Figure S3: Joinpoint regression analysis of the incidence density of carbapenem-resistant Gram-negative bacilli. Figure S4: Interrupted time-series analysis of changes in incidence density of other carbapenem-resistant Gram-negative bacilli. Figure S5: Joinpoint regression analysis of 14-day crude death rate. Figure S6: Joinpoint regression analysis of hand hygiene. Figure S7: Joinpoint regression analysis of complexity indicators associated with healthcare during the study period.
