# Supplementary material for: Impact of an Antimicrobial Stewardship Program on the Incidence of Carbapenem Resistant Gram-Negative Bacilli: An Interrupted Time-Series Analysis
Source: Antibiotics (Basel). 2021 May 16;10(5):586. doi: 10.3390/antibiotics10050586 (PMC8190633; doi:10.3390/antibiotics10050586)
Supplement: Supplementary file 1 [file antibiotics-10-00586-s001.zip › antibiotics-1214269-supplementary.pdf]

Supplementary material

***Supplementary Table S1*. Breakdown of monthly admitted patients**

Month	Total Patients	OBDs
Jan-2013	3194	23594
Feb-2013	3319	22799
Mar-2013	3427	24573
Apr-2013	3643	23946
May-2013	3545	23962
Jun-2013	3394	23886
Jul-2013	3278	22410
Aug-2013	2626	19133
Sep-2013	2771	19249
Oct-2013	3515	22783
Nov-2013	3339	22635
Dec-2013	3242	22275
Jan-2014	3445	23586
Feb-2014	3380	21642
Mar-2014	3540	23431
Apr-2014	3464	22006
May-2014	3537	23110
Jun-2014	3431	22414
Jul-2014	3325	22122
Aug-2014	2593	18786
Sep-2014	2954	20007
Oct-2014	3476	22558
Nov-2014	3492	23006
Dec-2014	3309	21452
Jan-2015	3340	23636
Feb-2015	3604	23524
Mar-2015	3744	23816
Apr-2015	3451	21960
May-2015	3448	22693
Jun-2015	3587	22453
Jul-2015	3291	21110
Aug-2015	2594	18845
Sep-2015	2928	19862
Oct-2015	3495	22215
Nov-2015	3437	22806
Dec-2015	3336	21627
Jan-2016	3026	23346
Feb-2016	3327	23333

OBDs: occupied bed days

***Supplementary Table S2*. Joinpoint regression analysis of antibiotic consumption**

DDDs/1000 OBDs	*Pre-intervention trend*<br><br>*MPC*<br>*[95% CI]*<br>*(p-value)*	*Point of change 1*<br><br>*Month*<br>*[95% CI]*<br>*(p-value)*	*Intervention trend 1*<br><br>*MPC*<br>*[95% CI]*<br>*(p-value)*	*Point of change 2*<br><br>*Month*<br>*[95% CI]*<br>*(p-value)*	*Intervention trend 2*<br><br>*MPC*<br>*[95% CI]*<br>*(p-value)*
Total antibiotics (ATC- J01)	-0.09%<br>[-0.62, 0.43]<br>(p=0.713)	17<br>[10, 33]<br>(p<0.05)	-0.74%<br>[-1.11, -0.36]<br>(p<0.001)	...	...
Carbapenems	-0.91%<br>[-2.80, 1.01]<br>(p=0.339)	13<br>[11, 14]<br>(p<0.05)	-28.10%<br>[-63.66, 42.24]<br>(p=0.331)	16<br>[14, 19]<br>(p<0.05)	-2.62%<br>[-4.06, -1.16]<br>(p=0.001)
Cephalosporins	2.59%<br>[1.49, 3.71]<br>(p<0.001)	16<br>[11, 28]<br>(p<0.05)	0.17%<br>[-0.39, 0.73]<br>(p=0.550)	...	...
β-lactams and β-lactamase inhibitors	-2.30%<br>[-3.07, -1.53]<br>(p<0.001)	11<br>[7, 13]<br>(p<0.05)	6.58%<br>[-6.04, 20.89]<br>(p=0.309)	14<br>[13, 22]<br>(p<0.05)	-0.05%<br>[-0.25, 0.15]<br>(p=0.606)
Quinolones	-0.71%<br>[-0.96, -0.46]<br>(p<0.001)	...	...	...	...
Aminoglycosides	-0.52%<br>[-1.03, -0.01]<br>(p=0.044)	...	...	...	...

MPC: monthly percentage change. CI: confidence interval

***Supplementariy Table S3*. Joinpoint regression analysis of the incidence density of carbapenem resistant-gram negative bacilli**

Incidence density	*Pre- intervention trend*<br>*MPC*<br>*[95% CI]*<br>*(p-value)*	*Point of change 1*<br>*Month*<br>*[95% CI]*<br>*(p-value)*	*Intervention trend 1*<br>*MPC*<br>*[95% CI]*<br>*(p-value)*	*Point of change 2*<br>*Month*<br>*[95% CI]*<br>*(p-value)*	*Intervention trend 2*<br>*MPC*<br>*[95% CI]*<br>*(p-value)*
**Global CR-GNB**	0.39%<br>[-2.91, 3.80]<br>(p=0.81)	14<br>[10, 17]<br>(p<0.05)	-6.06%<br>[-7.81, -4.27]<br>(p<0.001)	...	...
**Klebsiella pneumoniae**	4.58%<br>[-0.58, 10]<br>(p=0.081)	14<br>[10, 18]<br>(p<0.05)	-6.53%<br>[-8.91, -4.09]<br>(p<0.001)	...	...
**Other Enterobacteriaceae**	1.43%<br>[-1.63, 4.59]<br>(p=0.350)	15<br>[3, 18]<br>(p<0.05)	-18.89%<br>[-76.48, 179.66]<br>(p=0.732)	18<br>[8, 36]<br>(p<0.05)	-2.37%<br>[-5.33, 0.69]<br>(p=0.123)
**Escherichia coli**	3.55%<br>0.59, 6.59]<br>(p=0.019)	15<br>[3, 17]<br>(p<0.05)	-16.90%	18<br>[6, 36]<br>(p<0.05)	-2.56%<br>-5.42, 0.39]<br>(p=0.086)
**Proteus mirabilis**	-2.36%<br>[-3.79, -0.89]<br>(p=0.002)	...	...	...	...
**Klebsiella aerogenes/ Enterobacter cloacae**	-1.65%<br>[-2.35, -0.94]<br>(p<0.001)	...	...	...	...
**Pseudomonas aeruginosa**	-2.41%<br>[-3.82, -0.97]<br>(p=0.002)	...	...	...	...
**Acinetobacter baumannii**	-7.03%<br>[-8.78, -5.25]<br>(p<0.001)	...	...	...	...

CR-GNB: carbapenem-resistant gram negative bacilli. MPC: monthly percentage change. CI: confidence interval

***Suplementary Table S4*. Proportion of carbapenem-resistant *Escherichia coli* per year**

Year	Number of *E. coli* isolates	Number of CR-producing *E. coli*	Resistance proportion (%)
**2013**	393	24	6.1%
**2014**	355	20	5.6%
**2015**	326	10	3.1%
**2016***	36	2	5.6%

* Data for 2016 correspond only to the months included in the study period: January and February.

Supplementary Table S5. Interrupted time-series analysis of changes in hand hygiene compliance

Regression intercept	Pre-intervention trend	Change in Level[a]	Change in Trend[b]	Absolute effect[c]	Relative effect (%)[c]
67.14	-0.308	1.085	0.014	1.432	2.583
	(-0.576, -0.040)	(-1.145, 3.314)	(-0.240, 0.268)	(-6.657, 9.520)	(-12.48, 17.65)

Data are presented as monthly hand hygiene compliance with 95% confidence interval, unless otherwise specified. [a] Increase or decrease in the first month after the start of the intervention period with respect to the expected value. [b] Change in slope for the intervention period. [c] Absolute or percentage difference between the expected value according to the pre-intervention trend of hand hygiene compliance and the trend two years after the start of the intervention.

***Suplementary Figure S1*. Joinpoint regression analysis of carbapenem/ATC-J01 consumption**

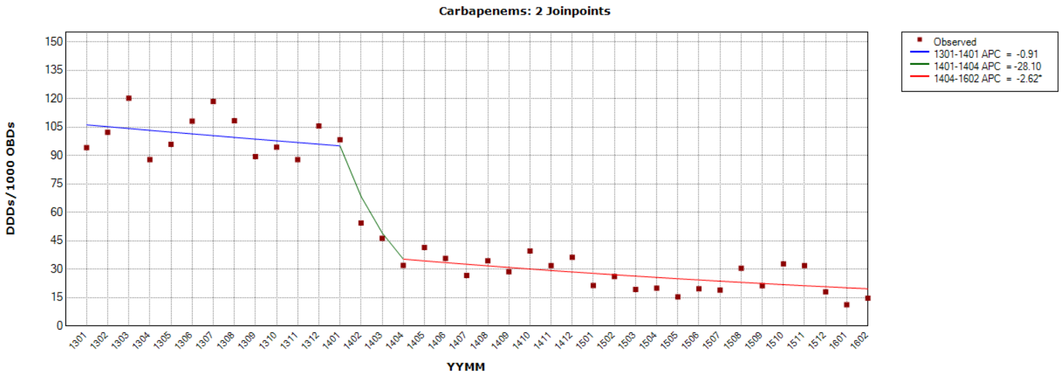

* Indicates that the Annual Percent Change (APC) is significantly different from zero at the alpha = 0.05 level.
Final Selected Model: 5 Joinpoints.

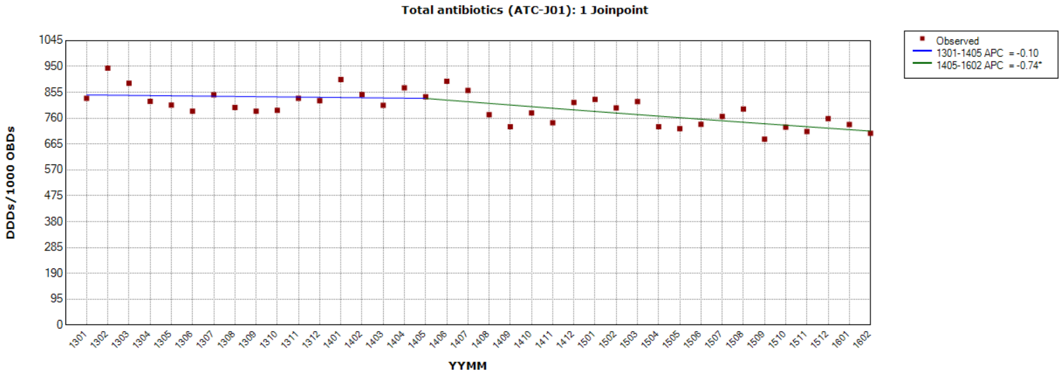

* Indicates that the Annual Percent Change (APC) is significantly different from zero at the alpha = 0.05 level.
Final Selected Model: 2 Joinpoints.

Date are presented as monthly defined daily doses (DDD) per 1000 occupied bed days (OBDs).

Supplementary Figure S2. **Interrupted time-series analysis of changes in consumption of other antibiotics**

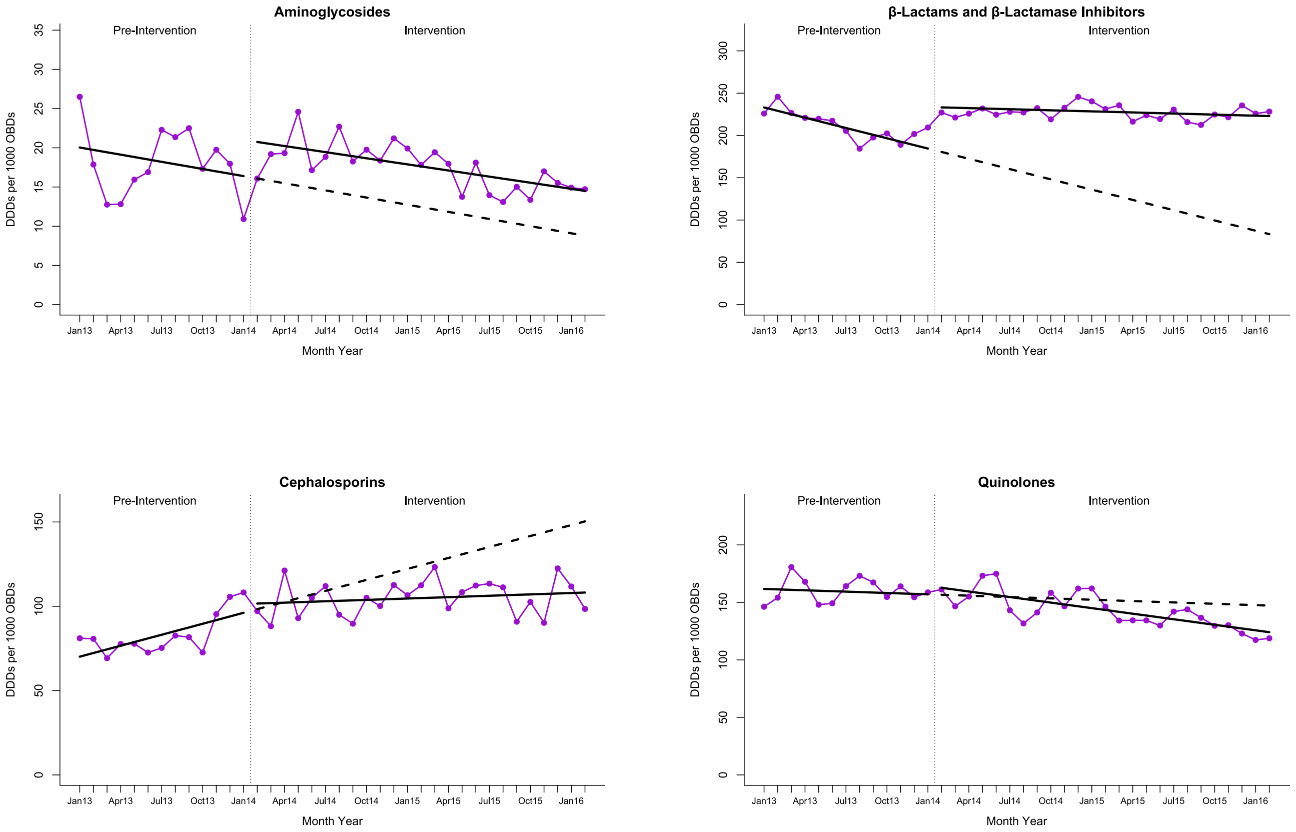

Date are presented as monthly defined daily doses (DDD) per 1000 occupied bed days (OBDs).
Solid purple line: antibiotic consumption time series. **Solid black lines**: pre-intervention and intervention trends.
Dashed black line: counterfactual (expected) trend

***Supplementary Figure S3*. Joinpoint regression analysis of the incidence density of carbapenem-resistant gram negative bacilli**

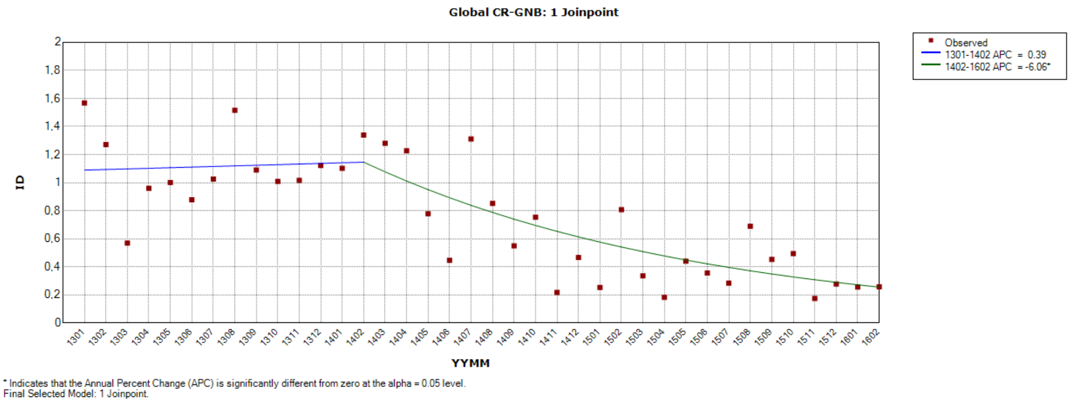

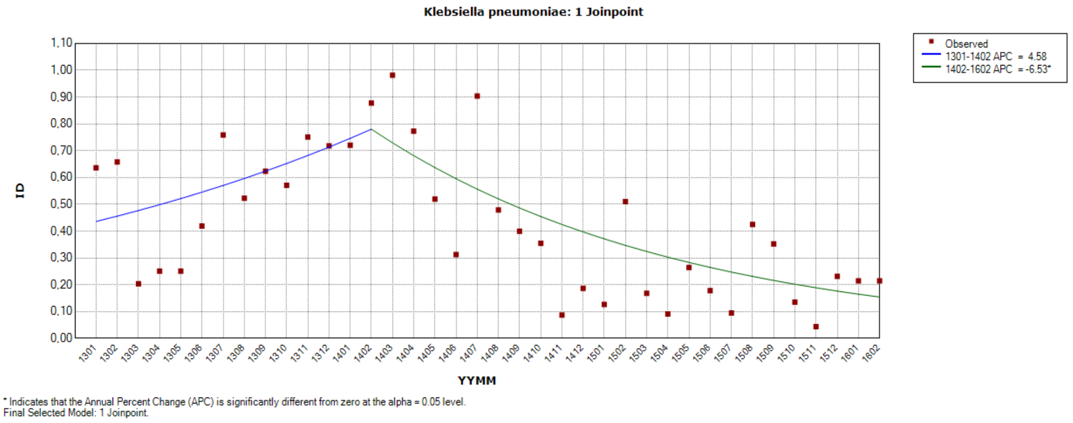

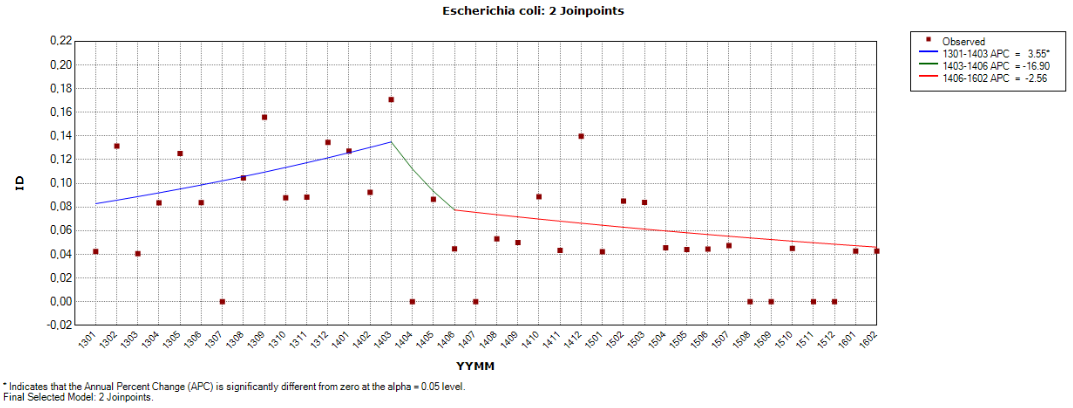

ID: Incidence density. YYMM: year, month

***Supplementary Figure S4*. Interrupted time-series analysis of changes in incidence density of other carbapenem-resistant Gram-negative bacilli**

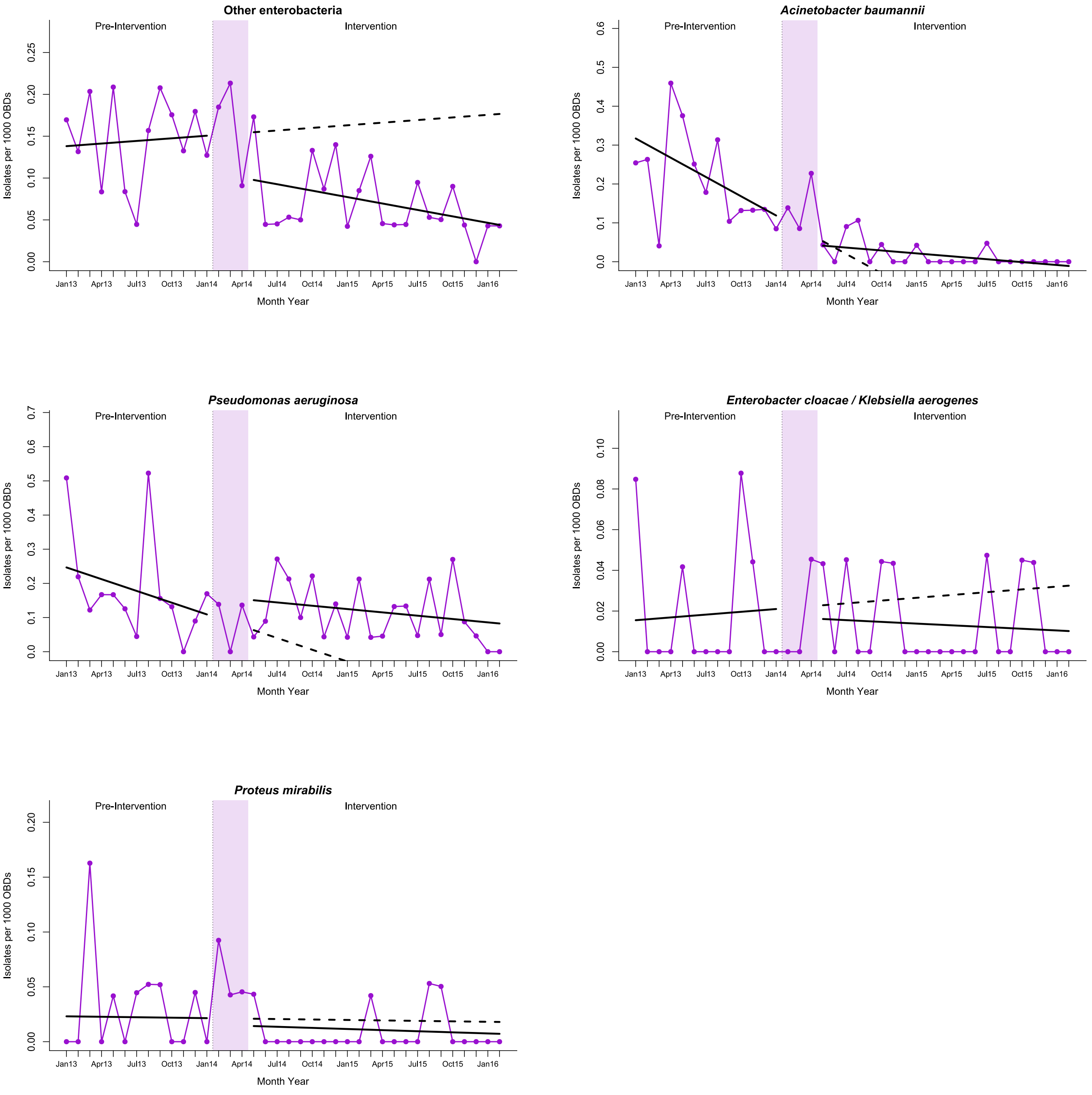

Date are presented as monthly isolates per 1000 occupied bed days (OBDs).
Solid purple line: CR-GNB incidence density time series. **Solid black lines**: pre-intervention and intervention trends. **Dashed black line**: counterfactual

***Supplementary Figure S5*. Joinpoint regression analysis of 14-day crude death rate**

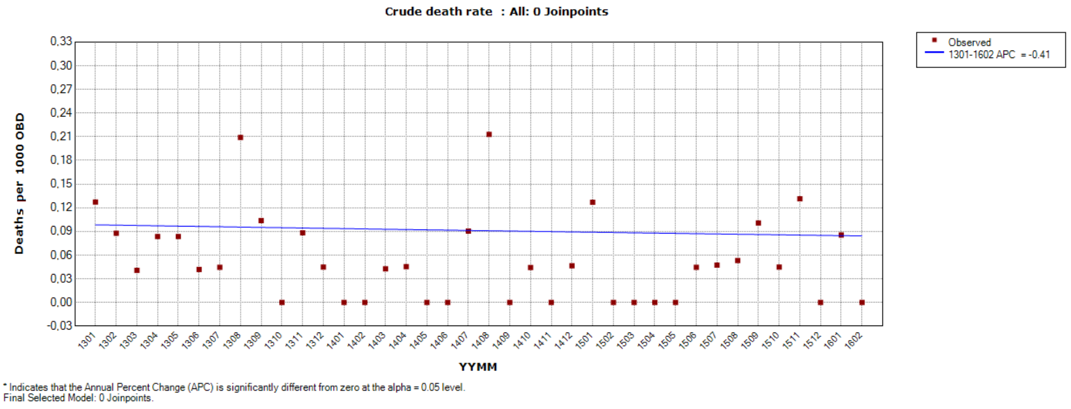

OBDs: occupied bed days

***Supplementary Figure S6*. Joinpoint regression analysis of hand higiene compliance**

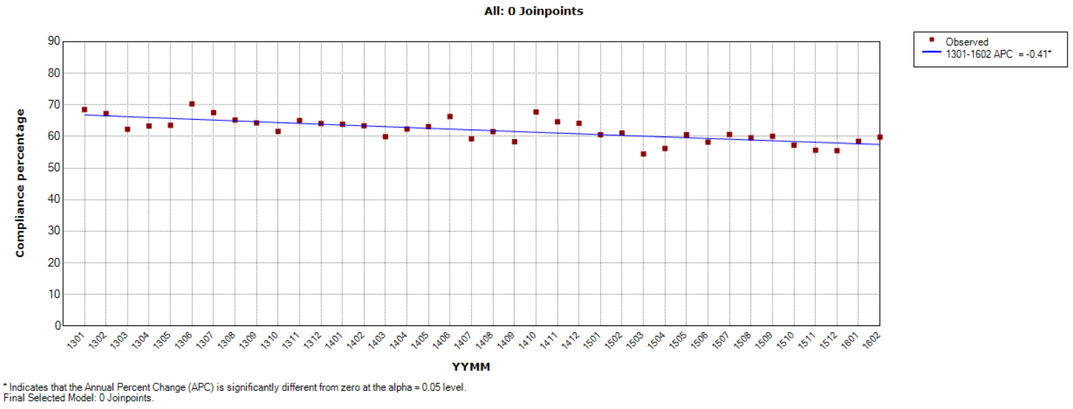

YYMM: year, month

***Supplementary Figure S7*. Joinpoint regression analysis of complexity indicators associated with healthcare during the study period**

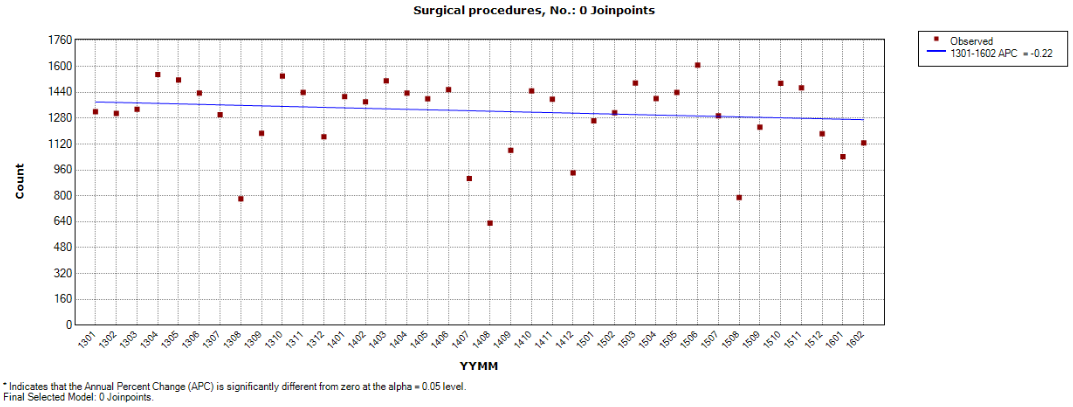

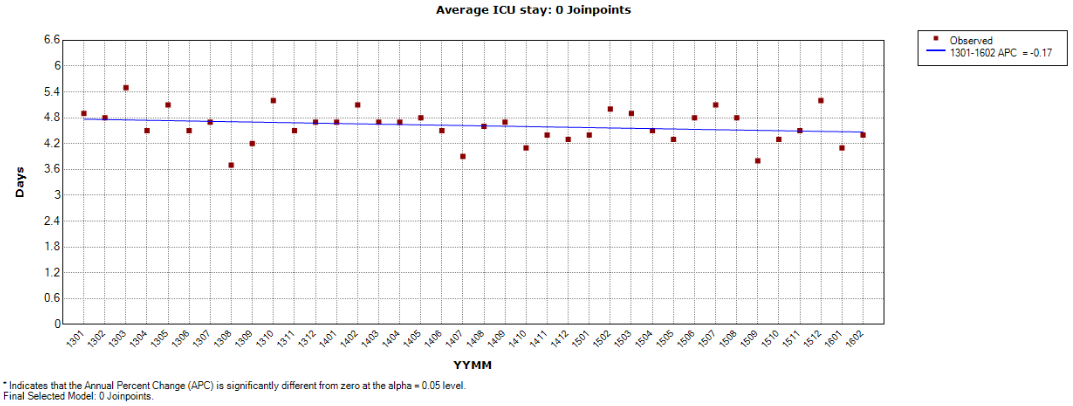

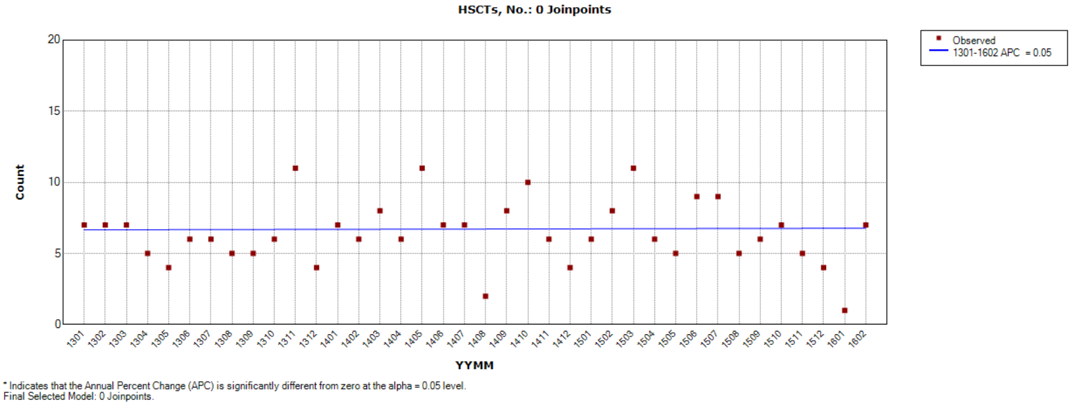

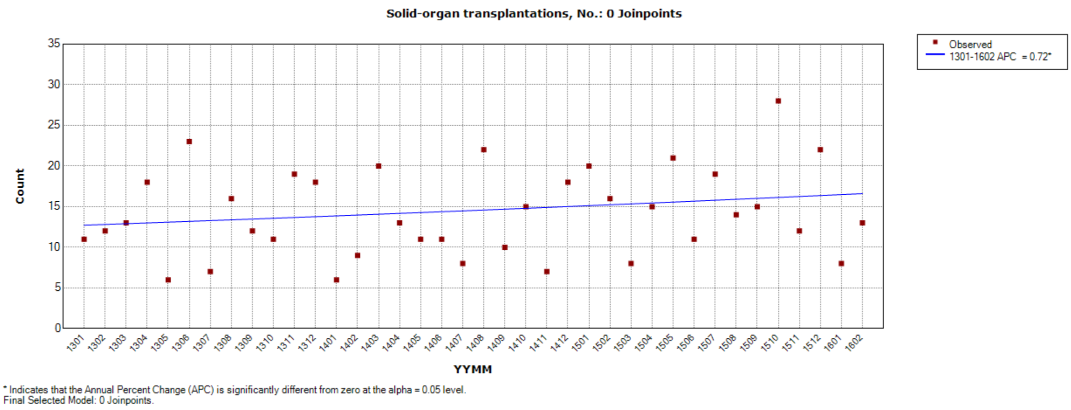

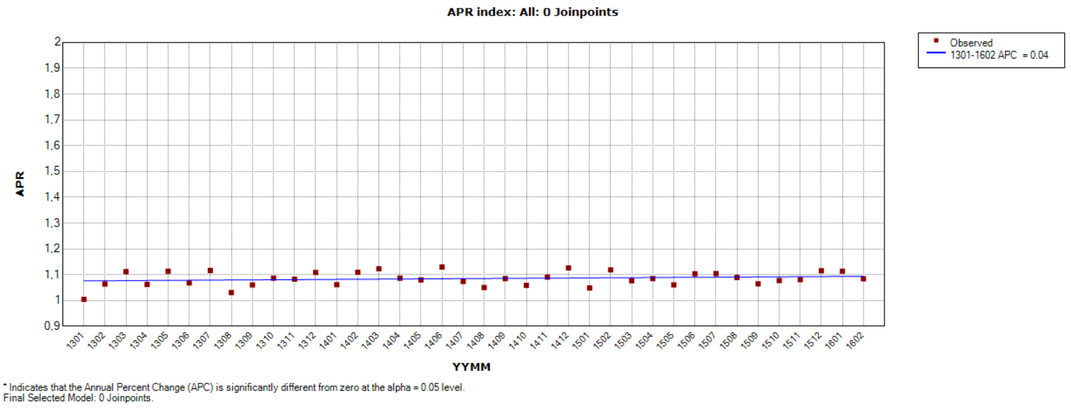

YYMM: year, month
